# Supplementary material for: Global hypo-methylation in a proportion of glioblastoma enriched for an astrocytic signature is associated with increased invasion and altered immune landscape
Source: eLife. 2022 Nov 22;11:e77335. doi: 10.7554/eLife.77335 (PMC9681209; doi:10.7554/eLife.77335)
Supplement: Figure 2—source data 1. [file elife-77335-fig2-data1.zip › Figure_2_source_data_1/Figure_2A/homerResults/motif6.similar.html]

motif6

## Information for motif6

T
A
C
G
A
G
T
C
G
T
A
C
G
C
A
T
A
T
G
C
A
G
T
C
C
G
T
A
T
A
G
C
G
T
A
C
C
G
T
A
C
T
A
G
A
T
C
G
  
Reverse Opposite:  

T
A
G
C
G
A
T
C
G
A
C
T
C
A
T
G
A
T
C
G
C
G
A
T
A
C
T
G
A
T
C
G
C
G
T
A
A
C
T
G
A
C
T
G
A
T
G
C
  

|  |  |
| --- | --- |
| p-value: | 1e-27 |
| log p-value: | -6.257e+01 |
| Information Content per bp: | 1.763 |
| Number of Target Sequences with motif | 28.0 |
| Percentage of Target Sequences with motif | 1.21% |
| Number of Background Sequences with motif | 2.2 |
| Percentage of Background Sequences with motif | 0.06% |
| Average Position of motif in Targets | 103.8 +/- 49.8bp |
| Average Position of motif in Background | 81.6 +/- 36.4bp |
| Strand Bias (log2 ratio + to - strand density) | -0.2 |
| Multiplicity (# of sites on avg that occur together) | 1.00 |
| Motif File: | file (matrix) reverse opposite |

### Similar de novo motifs found

|  |  |  |  |  |  |  |  |
| --- | --- | --- | --- | --- | --- | --- | --- |
| Rank | Match Score | Redundant Motif | P-value | log P-value | % of Targets | % of Background | Motif file |
| 1 | 0.609 | C A T G T C A G A T G C C G A T A C T G A T G C T C G A C T A G A T C G C G T A T C A G A T C G C T A G T C A G A C T G | 1e-24 | -55.604069 | 0.90% | 0.03% | motif file (matrix) |
| 2 | 0.604 | C G A T A C T G C T A G A C G T C A T G C A T G G A C T A C T G A C T G A C G T A C T G C A T G | 1e-16 | -38.831499 | 0.86% | 0.06% | motif file (matrix) |
